# Health service use and social vulnerability in a community-based sample of women on probation and parole, 2011–2013

**DOI:** 10.1186/s40352-015-0024-4

**Published:** 2015-06-19

**Authors:** Jennifer Lorvick, Megan L Comfort, Christopher P Krebs, Alex H Kral

**Affiliations:** 1 Behavioral and Urban Health Program RTI International, 351 California Street, Suite 500 San Francisco, CA 94104 USA; 2 Center for Justice, Safety and Resilience RTI International, 3040 East Cornwallis Road, HILL 412 Research Triangle Park, NC, 27709 USA

## Abstract

**Background:**

Most women involved in the criminal justice system are not incarcerated, but rather on probation or parole. We examined the receipt of health services and social vulnerability among women on parole or probation in the past year.

**Methods:**

In a community-based sample of 776 women who use crack cocaine or injection drugs, we compared those who had been on probation or parole in the past year with those who had no criminal justice involvement in the past year.

**Results:**

Women recently on probation or people were no more likely have health insurance, or to receive most health services, than women not in the criminal justice system. In addition, we found social vulnerabilities that contribute to poor health to be significantly more prevalent among women on probation or parole.

**Conclusions:**

There is a missed opportunity to address health and social needs of women on probation or parole.

## Background

Beginning in the mid-1980’s, the “War on Drugs” fueled a massive surge in the number of women involved in the criminal justice system for causes related to illicit drug use (Braithwaite et al. 2008). This population of women experiences high levels of infectious disease (Hammett and Drachman-Jones 2006; Nijhawan et al. 2011), chronic health conditions (Binswanger et al. 2010) and mental illness (Binswanger et al. 2010; Cloyes et al. 2010; Gunter et al. 2008). Scholars have analyzed how incarceration may serve as a “public health opportunity” for marginalized populations, because health care can be provided in correctional facilities in a context where basic needs such as food and shelter are met (Beckwith et al. 2010; Flanigan et al. 2010; Glaser and Greifinger, 1993). Often overlooked, however, is the fact that the vast majority of women in the criminal justice system are not behind bars, but rather on probation or parole (Philips 2012). Of the estimated 1.25 million women in the criminal justice system in 2012, over 80 % were on these forms of community supervision (Carson and Golinelli 2013; Maruschak and Bonczar 2013; Minton, 2013). Although the health-related needs of women on probation and parole may be similar to those of incarcerated women, the context of care is markedly different, as are the environments that shape health and illness (Belenko et al. 2004; Freudenberg et al. 2005).

Women on probation and parole are located at a unique social nexus: they must simultaneously manage correctional influences, which make them subject to institutional control and punishment; and community influences, which involve them in the risks and rewards of daily life in predominantly socially and economically disadvantaged communities. When women are under criminal justice supervision in the community, they continue to risk exposure to some of the strongest and best-known determinants of health disparities, such as poverty, substandard housing and lack of employment (Freudenberg et al., 2005; Hipp et al. 2010; Zierler and Krieger 1997). In addition, community settings present greater opportunities for potentially harmful health behavior such as drug use and unprotected heterosexual sex (Belenko et al. 2004).

Drug use is common among women involved in the criminal justice system, and drug-related violations are largely responsible for the increases in incarceration among women in recent decades (Greenfeld and Snell 2000). In a nationally representative sample of nearly 2000 women in jail, 59 % had drug use disorders. (Binswanger et al. 2010) Injection drug and crack cocaine use among women is associated with a number of serious but treatable health conditions, including HIV and sexually transmitted diseases (Latka et al. 2001; Shannon et al. 2008) It is in the interest of public health to facilitate access to health care for criminal justice involved women in community settings. (Binswanger et al. 2011) Among recently incarcerated women, the combined stigma of incarceration and drug use both increases their need for health services and makes these services harder to access (van Olphen et al. 2009).

The primary focus of probation and parole systems is public safety (Alameda County Probation Department 2014; U.S. Parole Commission 2014). However, consistent with system goals of rehabilitation and reducing recidivism, these systems have the potential to address health and social disadvantage. Institutional resources can be brought to bear to link women to services such as health care, housing assistance, and substance abuse treatment (Freudenberg et al. 2008). On the other hand, connection to a stigmatized system and having a record of criminal justice involvement may also exacerbate health disparities through social marginalization, instability and lack of economic opportunity (Blankenship et al. 2005; Dumont et al. 2013). In this paper, we examine potential harmful and beneficial impacts of probation and parole among women who use drugs in poor, predominantly African American neighborhoods of Oakland, CA (*N* = 756). Specifically, we assess how being on probation or parole is associated with receipt of health and social services, as well as how it may contribute to the physical and social vulnerability to poor health among impoverished women who use drugs.

## Methods

Data collection was conducted from July 2011 to July 2013 in Oakland, California. Located East of San Francisco, Oakland is a racially diverse, mid-sized city (population 400,000) in Alameda County. Most of the 15,000 probationers and parolees in Alameda County are clustered in 6 contiguous zip codes of Oakland (Urban Strategies Council 2012). These areas also have the highest proportions of African American residents and the most concentrated disadvantage in terms of health, income and housing (Alameda County Health Department 2013). Using targeted sampling techniques, (Kral et al. 2010; Watters and Biernacki 1989) we conducted recruitment from street settings in these areas. Eligibility criteria were crack cocaine or injection drug use in the 6 months prior to interview and age ≥18. We focused specifically on people who use injection drugs (PWID) and/or crack cocaine, because their is associated with HIV risk and infection (Booth et al. 1993). HIV testing and counseling and survey interviews were conducted at three easily accessible community field sites. Two of the sites were centrally located community churches, and the third was in a leased building near a major public transportation hub. An outreach worker recruited a total of 2424 total participants in neighborhoods surrounding the three sites. A participant screening instrument was used that included several items unrelated to eligibility criteria, in order to obscure eligibility requirements. Approximately 10 % of potential participants did not meet eligibility criteria.

Participants engaged in an informed consent process, a structured interview, HIV testing, and HIV pre- and post-test counseling. The quantitative interview was conducted face-to-face, with interviewers posing items verbally and recording responses in a computer-based personal interviewing system (Blaise®, Westat). Rapid testing for HIV infection was conducted using the OraQuick ADVANCE ® rapid HIV antibody test. Reactive results on the OraQuick test were confirmed with a second point-of-care test, the Clearview STAT-PAK®. Interview staff was trained in HIV testing and counseling as well as data collection techniques. All study procedures were reviewed and approved by a federally accredited Institutional Review Board. Participants received $20 remuneration for their contribution to the research, as well as referrals to medical and social services as appropriate.

In this analysis, we examined women in the sample who had been on probation or parole in the past 12 months (*n* = 202), and compared them with women with no criminal justice system involvement in the past 12 months (*n* = 565). Women who had been incarcerated but not on probation or parole (*n* = 91) were not included in the comparison group.

### Variables and measurement

We assessed the odds of two categories of outcome variables: physical and social vulnerability, and receipt of health and social services. Measures were drawn from the Urban Health Study (UHS) Survey Questionnaire, which has been used in over two dozen studies of people who use illicit drugs since the mid-1980’s (Kral et al. 2003). Time-frames for items in the UHS questionnaire were selected to optimize accurate recall and therefore vary somewhat by topic.

There were several outcomes related to physical and social vulnerability. Homelessness was defined as an affirmative response to the item “Are you currently homeless?” Physical assault was determined by a “yes” response to either item “In the past 12 months, has anybody punched, slapped, kicked, or physically hurt you?” Sexual assault was determined by a ‘yes’ response to the item, “In the past 12 months, has somebody used physical force or threats to make you have vaginal sex, anal sex, or oral sex with them?” The response option “illegal or potentially illegal sources” to the question, “In the past 30 days, what were your sources of money?” was used to determine illegal source of income. Age was determined by the item, “how old are you?” and cross-checked with self-reported year of birth. Injection drug use was assessed by the item, “In the past 6 months, have you injected drugs?” Drug injection was included as a physical vulnerability due to the large number of associated health risks.

Outcome variables regarding service utilization were determined by affirmative responses to items asking for the past 6 months “…have you been tested for HIV?” “…have you participated in drug treatment (excluding 12-step), “… have you received mental health care?”, “… have you received help from a professional or an agency to find a job?” To assist with recall, the survey was programmed to add calendar month to the timeframes, (e.g. “in the past 6 months, that is, *since January 2014…*”). Health insurance was determined by the item, “Do you have any health insurance?”

All analyses incorporated a central independent variable-probation or parole past year. This was defined as an affirmative response to the either question: “Have you been on probation in the past 12 months?” or “Have you been on parole in the past 12 months?”

### Analysis

Statistical analyses were conducted using SAS Version 9.2 (Cary, NC). Missing data were not imputed. Comparisons of dichotomous variables were conducted using a Pearson’s Chi-square test. Logistic regression analysis was used to identify whether probation or parole in the past 12 months was independently associated with the outcomes described above. All models were adjusted for age. In addition, each model was adjusted for potential confounding factors which were associated with the outcome at *p* ≤ 0.10 in bivariate analysis. Pearson correlation coefficients were examined for all the independent variables in the model. We set the level of statistical significance at *p* ≤ 0.05.

## Results

The sample was predominantly African American and impoverished (Table 1). Crack cocaine was the most commonly used drug. Even among the group of women with no criminal justice system involvement in the past year, lifetime involvement was high (Table 1). Exposure to the CJ system through family relationships was also common-42 % of women in each group reported having a family member who was currently incarcerated in jail or prison (data not shown).

**Table 1:** Characteristics of women on probation or parole past year compared to women with no criminal justice involvement

	Probation and parole (*n* = 202)	No CJ involvement (*n* = 564)
	Percent	Percent
**Race/ethnicity**		
African American	85	89
White	8	6
Latino	4	3
Other	3	2
**Drug use**		
Smoked crack past 30 days	89	89
Injected drugs past 30 days*	30	16
**Adult CJ history**		
Ever arrested*	100	81
Ever in jail*	99	80
Ever in prison*	34	18
Ever probation*	99	57
Ever parole*	31	17

*p < 0.05

Altogether, 174 women had been on probation, 17 had been on parole, and 11 had been on both in the past year. We found women on probation or parole had higher odds of physical and social vulnerabilities linked to health disparities (Table 2), compared to those with no CJ involvement in the past year. In multivariate analyses, we found women who had been on probation or parole in the past year had higher adjusted odds of homelessness, physical assault, sexual assault, illegal income and injection drug use in the past 6 months (Table 3).

**Table 2:** Social and physical vulnerability and receipt of services among women on probation or parole compared to women with no criminal justice involvement

	Probation or Parole past year (*n* = 202)	No CJ involvement past year (*n* = 564)	
	Percent	Percent	p.
Currently Homeless	60	38	<0.0001
Sexually assaulted past year	12	5	0.0022
Physically assaulted past year	36	22	0.0001
Illegal income past 6 months	44	20	<0.0001
Injected drugs past 6 months	34	18	<0.0001
**Services received past 6 months**			
HIV test	29	18	0.0022
Drug treatment	19	14	0.0883
Mental health care	31	28	0.4045
Job counseling	7	8	0.8684
Has health insurance	63	73	0.0103

Compared to women without criminal justice involvement in the past year, women on probation or parole had similar odds of having received drug treatment, job counseling, mental health care or health insurance (Table 4). HIV testing was the only service received more commonly among women who had been on probation or parole. Among the 55 women on probation or parole who had received HIV testing in the 6 months prior to interview, only 8 were tested in a correctional setting (jail, prison, probation or parole office). Most received testing at community-based agencies and outreach vans.

**Table 3:** Odds of social and physical vulnerability among women who were on probation or parole in the past year

Determinant (dependent variable)	Multivariate	
	AOR*	95 % CI
Model 1: Homeless*	2.2	1.6, 3.2
Model 2: Physically assaulted past year**	1.4	1.0, 2.1
Model 3: Sexually assaulted past year***	2.0	1.1, 3.7
Model 4: Illegal income past 6 mo’s^#^	2.4	1.6, 3.4
Model 5: Injected drugs past 6 months^##^	2.0	1.4, 2.9

*Adjusted for age, drug injection past 6 months, physical assault past 12 months, sexual assault past 12 months

**Adjusted for age, homelessness and sexual assault

***Adjusted for age, homelessness and physical assault

^#^Adjusted for age homelessness, injection drug use, physical assault and sexual assault

^##^Adjusted for homelessness

**Table 4:** Odds of receiving services among women who were on probation or parole in the past year

Services used (dependent variable)	Multivariate*	
	AOR	95 % CI
Model 1: HIV test past 6 months*	1.6	1.1, 2.5
Model 2: Drug treatment past 6 months**	1.3	0.8, 2.1
Model 3: Mental health care past 6 months***	0.9	0.6,1,3
Model 4: Job counseling past 6 months	0.7	0.4, 1.4
Model 5: Health insurance#	0.8	0.5, 1.1

*Adjusted for age, drug treatment past six months and current health insurance

**Adjusted for age, injected drugs past six months and health insurance

***Adjusted for age, injected drugs and health insurance

# Adjusted for age, drug treatment and drug injection

## Discussion

We found that past year involvement in the probation or parole systems was associated with higher levels of social and physical vulnerability among women who use drugs in a community-based sample. In addition, we found no association between probation and parole and receipt of supportive services such as substance abuse or mental health treatment. These findings suggest, that ties to the correctional system did not facilitate access to care and did not help address some of the basic social determinants of poor health, such as homelessness. Similarly, even though employment is a key stepping stone out of the criminal justice system (Bahr et al. 2010) job counseling was no more likely to be received by women on probation or parole than by other women in the sample. Women on probation and parole stand out as a particularly vulnerable subgroup in a population of women already experiencing severe challenges to health and wellbeing.

This research extends a small body of work regarding the health of women on probation or parole. Previous studies have found high levels of HIV risk (Adams et al. 2011a, 2011b) and cervical cancer risk (Belenko et al. 2004) among women on probation or parole, and widespread substance use among the subgroup with a history of physical or sexual victimization (Golder et al. 2014). In a nationally representative study, Vaughn (Vaugh et al. 2012) found that people on probation or parole in the past year were far more likely to have received alcohol treatment, drug treatment and mental health treatment in the past year, compared to those who had not been. These data were not stratified by sex. We found the opposite in our study, which used a more closely aligned comparison group, that is, women the same community who also used illicit drugs and were similarly vulnerable to criminal justice involvement. Our finding that women on community supervision were worse off than their counterparts is consistent with other work suggesting that vulnerability to poor health is exacerbated by incarceration (Dumont et al. 2013). This study is unique in comparing physical and social vulnerability and receipt of health services among women who were in the probation and parole system, compared to similar women who were not.

The absence of adequate planning to transition former inmates from correctional health care systems to community-based health systems has been noted frequently in research on correctional health (J. Adams et al. 2011a; Danzer, 2012; Freudenberg et al. 1998). For people in the community arm of the criminal justice system, the situation is similarly dire. In fact, for women who are assigned to probation without serving jail time, even correctional health care is unavailable. However, there is great potential for probation and parole systems to facilitate health care access through assistance for applications for Medicaid, which has been expanded in many states, including California, under the Affordable Care Act (ACA) (Rich et al. 2014; Rich et al. 2013). The ACA expanded eligibility for publicly-funded health care (Medicaid) to include single adults with incomes up to 133 % of the federal poverty level, a new development likely to benefit the participants in our study. Since the ACA was enacted midway through the study’s data collection period, it is possible that more women on probation and parole are now gaining health insurance access through these methods.

The findings of this study should be interpreted while recognizing several limitations. We were unable to assess whether there might be differential health needs in the two groups we compared, in which case differences in service utilization could be a sampling artifact. While both groups had essentially the same demographic characteristics, it is also possible that women on probation or parole had undetected similarities not related to their correctional involvement that influenced results. Like all self-report data, ours was subject to recall bias and social desirability, although the validity and reliability of self-report data from drug users has been verified (Darke, 1998). Finally, as this study was cross-sectional, it cannot shed light on the causal mechanisms for the associations we report here. The temporal relationship between the dependent and independent variables is not possible to determine. A more narrowly focused, longitudinal study is needed to appropriately examine how community corrections involvement may negatively or positively influence social conditions and service utilization among women who use drugs.

## Conclusion

The overriding theme of our findings is one of lost opportunities. Probation or parole involvement could provide a mechanism to connect a severely disadvantaged and vulnerable group of women to services that address basic needs (such as housing and legal sources of income) and health service needs (such as substance abuse and mental health treatment)-thereby promoting their health, stability and safety. Addressing these needs also would support the correctional objectives of reintegration and reduced recidivism. However, probation and parole systems are typically overtaxed and resources are not oriented towards goals that might be considered secondary to public safety (Taxman et al. 2009). Stronger alliances between probation and parole systems and community service agencies is one potential strategy to better meet the needs of women in the system (Dooris et al. 2013). In San Francisco, for example, the Adult Probation Office is collaborating with community agencies on a “one-stop re-entry center” to link people on probation with an array of stabilizing services (Community Corrections Partnership Executive Committee 2014). Established in 2013, no evaluations of this program are available at this time.

Our research indicates that, currently, women on probation or parole are more imperiled than their counterparts who are not affiliated with the criminal justice system, with a higher prevalence of homelessness and violent victimization. More in-depth research is needed to understand why this is the case. Concurrently, probation or parole involvement should be better leveraged as a tool to improve the health and well-being of impoverished women who use drugs.
